# Willingness of the UK public to volunteer for testing in relation to the COVID-19 pandemic

**DOI:** 10.1186/s12889-022-12848-z

**Published:** 2022-03-22

**Authors:** Tushna Vandrevala, Amy Montague, Philip Terry, Mark D. Fielder

**Affiliations:** 1 Centre for Applied Health and Social Care Research, Faculty of Health, Social Care and Education, Kingston University and St George’s University of London, London, UK; 2 Department of Psychology, Kingston University London, London, UK; 3 Department of Biomolecular Sciences, School of Life Sciences, Pharmacy and Chemistry, Faculty of Science, Engineering and Computing, Kingston University, Kingston-Upon-Thames, UK

**Keywords:** COVID-19, Testing, Risk perception

## Abstract

**Background:**

The World Health Organization declared the rapid spread of COVID-19 around the world to be a global public health emergency. The spread of the disease is influenced by people’s willingness to adopt preventative public health behaviours, such as participation in testing programmes, and risk perception can be an important determinant of engagement in such behaviours.

**Methods:**

In this study, we present the first assessment during the first wave of the pandemic and the early stages of the first UK lockdown in April & May 2020 of how the UK public (*N* = 778) perceived the usefulness of testing for coronavirus and the factors that influence a person’s willingness to test for coronavirus.

**Results:**

None of the key demographic characteristics (age, gender, education, disability, vulnerability status, or professional expertise) were significantly related to the respondents’ willingness to be tested for coronavirus. However, closely following the news media was positively related to willingness to be tested. Knowledge and perceptions about coronavirus significantly predicted willingness to test, with three significantly contributing factors: worry about the health and social impacts to self and family; personal susceptibility; and concerns about the impacts of coronavirus on specific demographic groups. Views on testing for coronavirus predicted willingness to test, with the most influential factors being importance of testing by need; negative views about widespread testing; and mistrust in doctor’s advice about testing.

**Conclusions:**

Implications for effective risk communication and localised public health approaches to encouraging public to put themselves forward for testing are discussed. We strongly advocate for effective communications and localised intervention by public health authorities, using media outlets to ensure that members of the public get tested for SARs-CoV2 when required.

**Supplementary Information:**

The online version contains supplementary material available at 10.1186/s12889-022-12848-z.

## Background

In December 2019 a novel coronavirus emerged as a cause of a pneumonia in Hubei province of China with an apparent epicentre in the city of Wuhan [1]. The new coronavirus (SARS-CoV-2) causes acute respiratory syndrome (COVID-19). The disease has subsequently spread globally and was declared a pandemic by the World Health Organization (WHO). Studies have consistently noted that men appear to be at greater risk to the infection than women [2] ethnicity was linked to the acquisition [3–5] and death rates appear to increase following SARs-CoV2 in patients with an increased age [6–9].

In 2020, the pandemic response in many countries, including the UK, has been to “lock down” communities to prevent further spread of the virus, which caused economic hardship and other negative societal effects on the affected communities [2]. Effective and extensive testing regimes have been essential to the successful lifting of lockdown and have a role to play in preventing future lockdowns [10, 11] and continue to be the corner stone of public health response to the global pandemic. An approach based on testing and isolating has been extensively adopted in some countries with great effect. For example, in the early stages of the pandemic, South Korea saw dramatic falls in cases by implementing an extensive testing regime supported by a trace-and-quarantine program for the contacts of those testing positive [12].

The two main types of test currently in use are a molecular test that detects the RNA genome of the SARs-CoV-2 virus using an RT-PCR test protocol and a serology-based, and tests for the presence of an immune response to the virus, indicating whether a person has had the virus previously. These are clearly useful epidemiological tools, not only for understanding the extent of the outbreak, but also for estimating the prevalence of asymptomatic cases in the community [13].

At the time of data collection (April–May 2020) the importance of identifying asymptomatic individuals were still unclear [14], while in recent months regular RNA testing has been imperative in the UK strategy of “living with the virus” and avoiding subsequent lockdowns. Initial reservations towards using mass asymptomatic testing or community testing as a tool to help bring the epidemic rapidly to an end included distribution and concerns about engaging hard-to-reach groups and about accessibility and equity issues [15]; However, this has enabled those that test negative to return to ‘normal’ life, whilst those who test positive can be treated and their contacts quarantined. In these early stages, there were some who saw this approach as too intrusive and authoritarian. Indeed, the WHO’s standard containment approach of “find, test, treat, and isolate” was not the approach advocated in the UK at the time of data collection, and community-based case identifying, testing, and contact tracing were not common practice.

The current study was the first UK based study that sought to understand public perceptions of personal and societal risk factors towards coronavirus during the first wave and early stages of lockdown in the UK and examined how these perceptions influenced personal willingness to be tested for COVID-19. Understanding public perceptions of personal and societal risk factors during pandemics and how they can influence the public’s willingness to cooperate and adopt health-protective behaviours during pandemics was essential at a time where these behaviours were not social norms [16]. However, compared to other risk domains, such as environmental risks far less was known about how the public perceives risks associated with emerging infectious diseases [17, 18]. The public’s capacity to comply with recommendations during pandemics or other emergencies was influenced by a range of modifiable or non-modifiable factors, including their sociodemographic status; what they perceive to be their own susceptibility to infection [19]; whether they perceive the infection to be serious; whether they have the necessary confidence to make changes; and the perceived costs and benefits of actions [16, 18]. In order to develop effective public health messages it was therefore important to understand the public’s perceptions of the threat posed by the pandemic and how they are likely to respond behaviourally.

When applied to testing or screening, the use of theory to explain health behaviour and inform interventions has been found to have benefits [20]. The most commonly cited theories in this context are the Health Belief Model (HBM) and the Protection Motivation Theory, PMT [19, 21]. The HBM uses susceptibility, severity, costs, benefit, cues to action, health motivation and perceived control to predict likelihood of behaviour. The Protection Motivation Theory (PMT) applies general social cognitive constructs of severity, susceptibility, response effectiveness, self-efficacy and fear to health contexts to explain behavioural intention [19]. HBM and PMT constructs have been effective in predicting the uptake of testing, and the model has been used to inform interventions for non-communicable diseases, primarily cancer [22, 23]. Of all elements, perceived barriers have been shown to be the most predictive of actual uptake, whilst research suggests that external cues to action can be manipulated to reduce barriers [24]. Some studies show that perceived response and self-efficacy were not good predictors to behavioural intention [25]. This evidence, however, cannot easily be extrapolated to infectious diseases, which include symptomatic testing and additionally involve the potential of becoming a disease carrier, adding a potential component of stigma [26].

Therefore, we carried out a cross sectional online survey of public beliefs about and attitudes towards coronavirus and testing for coronavirus during April & May 2020 during the first wave and early stages of lockdown in the UK. Our study examined how these perceptions influenced personal willingness to be tested for COVID-19. During this time community testing was not commonplace and covid testing was limited to hospital settings. The primary aim of the current study was to identify factors that increase or decrease willingness to volunteer for testing.

## Methods

### Participants

The opportunistic sample comprised 778 UK residents (181 males and 589 females, 8 other/prefer not to say); their ages ranged from 18 to 80 yrs. old, with the average age being 47 yrs. The sample was predominantly white (86%), with remaining participants identifying as ‘Black’, ‘Asian’, ‘Mixed’ and ‘other’ ethnic groups (14%). Full demographic details are given in Table 1. At the time of completing the survey, only 3.5% of the sample had been tested for coronavirus.

**Table 1:** Demographic characteristics of sample (*N* = 778)

***Participant Characteristic***	***Number of participants***	***% within total participants***
Sex		
Male	181	23.5
Female	589	76.5
Age (yrs)		
Under 45	324	41.6
45–69	406	52.2
70 and above	44	5.7
Missing	4	0.5
Ethnicity		
White	675	86.8
Black	9	1.2
Asian	54	6.9
Mixed	22	2.8
Other	18	2.3
Highest educational Qualification		
Post-Graduate	284	36.5
Graduate	257	33
A-levels	123	15.8
GCSE’s	62	8
NVQ	6	0.8
None	20	2.6
Other	26	3.3
Disability (hidden or visible) or long-term illness		
Yes	145	18.6
No	632	81.2
Missing	1	0.1
Government-defined ‘vulnerable group’		
Yes	32	4.1
No	731	94
Not applicable	14	1.8
Missing	1	0.1
Tested for Coronavirus		
Yes	27	3.5
No	751	96.5
Professional Expertise		
Health	75	9.6
Social Care	19	2.4
Scientist	99	12.7
Key worker	78	10
Multiple	68	8.7
None mentioned	439	56.4
Perceived level of scientific knowledge		
Advanced and above	196	25.2
Average	444	57.1
Poor and below	137	17.6
Missing	1	0.1

### Materials

Participants completed questionnaires that were developed specifically for this study (see Additional file 1). These questionnaires addressed “Knowledge and perceptions about coronavirus” and “Views on testing for coronavirus”. The respondents indicated their level of agreement with each statement on a 4-point Likert scale from ‘strongly disagree’ to ‘strongly agree’. Based on the initial responses, factor analysis consolidated the variables into discrete, coherent sub-scales (see Additional file 2 for further details). Scores from these scales were the primary variables in all subsequent analyses (see Additional file 3 for breakdown of variables).

Part 1: Knowledge and Perceptions about coronavirus included the following sub-scales:

*Confidence that taking action is effective* (4 items, α = .651) reflects a person’s confidence in actions being effective in terms of protecting themselves and others from coronavirus.

*Perceived severity and threat* (5 items, α = .793) encompasses the participant’s beliefs about the ability of coronavirus to cause severe health problems or to represent a serious threat to them or to others.

*Personal susceptibility* (3 items, α = .776) denotes the extent to which participants perceived themselves to be at risk of coronavirus due to their health status or age.

*Worry about economic implications* (3 items, α = .696) reflects participant concerns about personal finances and the long-term impacts of the virus on their job prospects and the economy.

*Impact of coronavirus on specific demographic groups* (5 items, α = .686) indicates the participant’s perceptions about the particular impacts on coronavirus on people over the age of 70 years old, ethnic communities, and/or people with underlying health conditions.

*Positive impacts on self and society* (2 items, α = .717) encompasses beliefs that the virus has had a positive impact on the participant’s life and will have a positive impact on society in the future.

*Worry about the health and social impacts on self and family* (4 items, α = .452) indicates the extent to which the participant worries about contracting coronavirus and its likely impacts on self and family.

Part 2: Views on testing for coronavirus included the following sub-scales:

*Negative views about widespread testing* (4 items, α = .847) denotes whether widespread testing was considered by participants to be a waste of time and resources.

*Importance of testing “by need”* (5 items, α = .814) indicates how important participants felt it was to prioritise testing for themselves if they show symptoms, or for vulnerable people or those who work with vulnerable people.

*Testing considered as an effective protective measure* (4 items, α = .804) reflects the extent to which participants believe that testing could protect them or others from being infected by the coronavirus.

*Trust in government approach to testing* (4 items, α = .795) indicates how much the participant trusts the government’s COVID testing strategy.

*Willingness to be tested* (2 items, α .814) indicates the extent to which participants would consider getting tested for coronavirus.

*Trust in doctor’s advice about testing* (3 items, α = .576) indicates whether participants trust their doctors to inform them if they needed to get tested.

*Beliefs that testing can indicate immunity* (2 items, α = .554) indicates whether participants believe testing will tell them if they have immunity from coronavirus and whether they have had coronavirus previously.

*Worries about testing outcome* (2 items, α = .556) reflects the participant’s worries about the results of coronavirus testing, including being a future burden on their family.

### Procedure

Participants were recruited via an online advertisement distributed through social media during the UK government-mandated ‘coronavirus lockdown’ period between April 26 and May 15, 2020. Participants aged 18 years and above were invited to take part in a study looking at attitudes towards coronavirus and testing during the pandemic. Participants could access a hyperlink in the advertisement which directed them to the anonymous online survey. The survey was presented via the Qualtrics platform. Study information and consent forms were presented before the first survey questions (demographic characteristics) and then the subsequent coronavirus-related questions. It took participants approximately 15–20 min to complete and a debrief sheet was provided when they finished. The study received a favourable ethical opinion from the Kingston University Research Ethics Committee.

Several statistical analyses were carried out on the study data. Comparisons between independent groups were conducted using T-test. However, for some analyses due to the number of tied data values, non-parametric test Spearman’s Rho was also used to assess associations between variables. Finally, multiple regressions were conducted to determine factors which were significant predictors of a given dependent variable. All these analyses were conducted using SPSS v26.

## Results

None of the key demographic characteristics was significantly related to participant’s willingness to be tested for coronavirus. Thus, there were no differences between men and women or between white and BAME respondents in terms of willingness to be tested (respectively: t = 0.236, df = 730, *p* = .813; t = 0.979, df = 735, *p* = .328). Similarly, age did not predict willingness to be tested (Spearman’s rho = 0.052, *p* = .161; *N* = 734). People who worked in professions that might confer relevant expertise (e.g. healthcare worker, social worker; *N* = 413) did not differ from those who worked in other fields (*N* = 319; t = 0.436, df = 730, *p* = .663); self-declared level of scientific knowledge was also unrelated to willingness to be tested (Spearman’s rho = 0.008, *N* = 737, *p* = .820), and level of education also failed to predict willingness to be tested (F[6730] = 1.243, *p* = .282). However, the extent to which a person closely followed the news was modestly positively correlated significantly with willingness to test (Spearman’s rho = 0.203, *N* = 734, *p* < 0.001). Finally, neither self-declared vulnerable status nor disability status increased a person’s willingness to be tested in comparison with non-vulnerable or non-disabled respondents (respectively: t = 0.518, df = 730, *p* = .605; t = 0.508, df = 734, *p* = .612).

By multiple regression, the sub-scales from the knowledge and perceptions about coronavirus section of the survey together significantly predicted willingness to test (F[7727] = 8.680, *p* < 0.001) but they explained only 7.7% of the variance in scores for willingness to be tested. Individual factors that significantly contributed to the regression model were “worry about the health and social impacts to self and family” (standardized beta = 0.120, *p* = .003), “personal susceptibility” (standardized beta = − 0.100, *p* = .014) and “impacts of coronavirus on specific demographic groups” (standardized beta = 0.096, *p* = .012). Factors that did not significantly contribute were “perceived severity and threat” (*p* = .064), “confidence that taking action is effective” (*p* = .606), “worry about economic implications” (*p* = .537) and “positive impacts on self and society” (*p* = .458).

Looking at the second part of the survey (views on testing for coronavirus), all of the factors together strongly predicted “willingness to be tested” (F[7724] = 29.823, *p* = < 0.001) and explained 22.4% of the variance in scores. The most influential factors were “importance of testing “by need”” (standardized beta = 0.270, *p* < 0.001); “negative views about widespread testing” (standardized beta = − 0.233, *p* < 0.001); “trust in doctor’s advice about testing” (standardized beta = − 0.081, *p* = .015). The negative result denotes that participants were less likely to trust their doctor’s advice about testing while making decisions regarding their willing to test. The non-significant factors in the model were “testing considered as an effective protective measure” (*p* = .144); “trust in government approach to testing” (*p* = .522); “beliefs that testing can indicate immunity” (*p* = .061) and “worries about testing outcome” (*p* = .517).

## Discussion

Testing for SARs-CoV2 enables infected people to be identified and isolated in order to reduce the spread of the virus and can allow for contact tracing of potentially exposed individuals and yields data on localised rates of infections that can inform public health interventions. In the early stages of the pandemic response, there was some evidence on how clinicians use and interpret SARs-CoV2 tests [27], but little was known about how the public perceive risk regarding coronavirus [17, 18]. There was no research on how a person’s demographic characteristics or views and knowledge about testing might influence their willingness to be tested. In a situation such as the current pandemic it is of utmost importance to understand public perception of risk, their knowledge about key healthcare interventions, and their willingness to engage in testing. Our study has highlighted that in the context of coronavirus testing, previous social cognitive models (such HBM, PMT) have some utility in predicting intention to test, but fail to take into account more contextual factors, such as worry about impact on self and family members, assessment of vulnerable status, trust in doctors and how news might influence intention to test. Perhaps future theories to understand and predict covid protecting behaviours, such as coronavirus testing need to account for a range of contextual factors beyond those included in social cognitive theories.

In the early days of testing, it was recognised that as lockdown eases and test and trace systems would be seen by public health and government officials as a quick, vital and effective way to control the spread of the virus, the findings presented in this paper have potentially important implications. The current survey results indicated that willingness to be tested for coronavirus was not related to key demographic variables, such as age, gender, ethnicity, educational status or to professions that might confer relevant expertise (e.g. healthcare worker, social worker) or to self-declared level of scientific knowledge. Neither self-declared vulnerable status nor disability status influenced a person’s willingness to be tested. Willingness to be tested was unrelated to educational attainment, professional expertise or self-declared level of medical knowledge. Moreover, the findings clearly demonstrate that willingness to be tested is strongly influenced by their perceptions towards coronavirus. Specifically the extent to which members of the public worry about contracting coronavirus and its likely impacts on self and family and the extent to which they perceive themselves to be at risk of coronavirus due to their health status or age and their perceptions about the particular impacts on coronavirus on people over the age of 70 years old, ethnic communities and/or people with underlying health conditions. Despite this, perceptions of the overall severity and threat posed by the pandemic in the early stage of the pandemic where community testing was not the norm did not influence willingness to be tested; it seems that the threat (and likelihood of action) was viewed through the lens of personal circumstance. Willingness to be tested was therefore not a straightforward decision. Moreover, how people perceive the risk is not necessarily related to the actual risk; nevertheless, it is the perceived risk that influences protective behaviours [28]. Uncertainty about the situation and perceived exaggeration have been associated with a reduced likelihood of implementing recommended protective behaviours during previous pandemics [15].

Encouragingly, the findings of the current study suggest that the public’s views on vulnerability predicted willingness to be tested. Increased willingness was associated with beliefs that it was important to prioritise testing “by need” for themselves (if symptomatic) or for vulnerable people, or for those working with vulnerable people. Test intention during the early stages of the pandemic was negatively related to beliefs that widespread (indiscriminate) testing would be a waste of time and resources. Furthermore, the public were also less likely to report willingness to be tested if they trusted their doctors to advise them about the need for testing. Interestingly, our findings suggest that the extent to which a person closely followed the news were related to willingness to test, albeit that a single question relating to generic news exposure limits our ability to interpret the kinds of news that are most important or the extent to which a person objectively engages with news media However, it is not unusual for research to probe media exposure in this way, with other emerging COVID-19 research also using broad measures of self-reported engagement with COVID-19-related news to assess associations with mental health vulnerabilities [29, 30]. The influence of the media (in contrast to mistrust in doctors to advise them about testing) warrants further investigation: for example, future studies should examine whether specific sources of media consumption (social media versus tabloid, mainstream news) were particularly impactful on perceptions of coronavirus and testing. In contrast, the following did not predict intention to test: the extent to which the respondent felt that testing was an effective protective measure; their beliefs about testing providing immunity; their worries about testing outcomes; and their trust in the government’s approach to testing. The latter outcome contrasts with previous research indicating that trust in government is related to risk perceptions [17].

The current study covers a large cross-sectional sample of the adult population in the UK. Members of the public were excluded from the sample if they were unable to communicate in English and it is duly noted that this exclusion may have had an effect on the representation of some ethnic minority groups or communities. Due to the use of opportunity sampling, the sample was over representative of females. In addition, participation in the current study was on a voluntary basis. As a result, there is potential for the participants in this study to show a level of self-selection based upon their particular concern with regard to this pandemic.

## Conclusions

The findings presented here advocate that public views on willingness to test were related to modifiable factors, such as risk perceptions to coronavirus and views on testing. In order to develop effective public health messages, interventions should be designed to address these specific public perceptions posed by the pandemic, which, in turn correspond to how people respond behaviourally. There is, therefore, a growing need to ensure that a public health approach towards case test and tracking, with the adequate support of public health teams who know their communities, the nature of the outbreak in their communities is employed to assist individuals and communities navigate some of the decisions they face regarding testing. Effective communications and localised intervention by public health authorities, using media outlets to disseminate information are of particular use in ensuring that members of the public get tested for SARs-CoV2 when required. Scally [31], advocates that plans for community contact tracing must be adequately resourced, decentralised, and led by local public health teams; Public Health England and the NHS must fully support these plans. A localised public health approach could specifically address the public’s risk perception of the virus and testing and ensure than public messaging is reframed to address these issues.

It is critical to understand the public’s risk perception in relation testing. This should be bought into sharp focus given the clear need for ongoing testing as the pandemic progresses, this is especially true with the emergence new variants of the virus. It is well known that as viruses replicate, they mutate [32]. This has been seen with the emergence of the Delta variant “Delta plus” (AY.4.2), and more recently the Omicron variant. As such, continued testing and the acceptance of such testing by the public will be critical in the ongoing attempts to understand infection patterns and bring the pandemic under control.

## Supplementary Information

**Additional file 1.** Questionnaire. Knowledge and Perceptions about Coronavirus items with scale points. Views on Testing for Coronavirus items with scale points.

**Additional file 2. **Factor Analysis. Table 3 and 4: Factors with loadings for Knowledge and perceptions about Coronavirus *&* Factors with loadings for Views on Testing for Coronavirus.

**Additional file 3.** Table 2. Means, standard deviations, Cronbach’s alpha scores and list of items, for each subscale.

## Data Availability

The datasets used and analysed during the current study are available from the corresponding author on reasonable request.

## Abbreviations

SARS-CoV-2: Severe Acute Respiratory Syndrome Coronavirus 2
COVID-19: Coronavirus Disease 2019
WHO: World Health Organisation
BAME: Black, Asian and Minority Ethnic
RT-PCR: Real-Time Polymerase Chain Reaction
RNA: Ribonucleic Acid tests
GP: General Practitioner

## References

[1] World Health Organisation. Novel coronavirus (2019-ncov) situation report – 1. 2020. Available from: https://www.who.int/docs/default-source/coronaviruse/situation-reports/20200121-sitrep-1-2019-ncov.pdf?sfvrsn=20a99c10_4. Accessed 14 Mar 2022.

[2] Caramelo F, Ferreira N, Oliveiros B. Estimation of risk factors for COVID-19 mortality - preliminary results. MedRixc. 2020. 10.1101/2020.02.24.20027268.

[3] Pareek M, Bangash M, Pareek N, Pan D, Sze S, Minhas J (2020). Ethnicity and COVID-19: an urgent public health research priority. Lancet..

[4] Pan D, Sze S, Minhas J, Bangash M, Pareek N, Divall P (2020). The impact of ethnicity on clinical outcomes in COVID-19: a systematic review. EClinicalMedicine..

[5] Rimmer A (2020). Covid-19: disproportionate impact on ethnic minority healthcare workers will be explored by government. BMJ..

[6] Garg S, Kim L, Whitaker M, O’Halloran A, Cummings C, Holstein R (2020). Hospitalization rates and characteristics of patients hospitalized with laboratory-confirmed coronavirus disease 2019 — COVID-NET, 14 states, march 1–30, 2020. MMWR Morb Mortal Wkly Rep.

[7] Wang L, He W, Yu X, Hu D, Bao M, Liu H (2020). Coronavirus disease 2019 in elderly patients: characteristics and prognostic factors based on 4-week follow-up. J Inf Secur.

[8] Armitage R, Nellums L (2020). COVID-19 and the consequences of isolating the elderly. Lancet Public Health.

[9] Mahase E (2020). Covid-19: death rate is 0.66% and increases with age, study estimates. BMJ.

[10] Scientific Advisory Group for Emergencies (SAGE). Consensus view on behavioural and social interventions. 2020. Available from: SPI-M-O: Consensus view on behavioural and social interventions - 16 March 2020 (publishing.service.gov.uk).

[11] Peto J, Alwan NA, Godfrey KM (2020). Universal weekly testing as the UK COVID-19 lockdown exit strategy. Lancet..

[12] Cohen J, Kupferschmidt K (2020). Countries test tactics in ‘war’ against COVID-19. Science..

[13] Burki T (2020). Testing for COVID-19. Lancet Respir Med.

[14] Treibel TA, Manisty C, Burton M (2020). COVID-19: PCR screening of asymptomatic health-care workers at London hospital. Lancet..

[15] Peto J (2020). Covid-19 mass testing facilities could end the epidemic rapidly. BMJ.

[16] Rubin G, Amlot R, Page L, Wessely S (2009). Public perceptions, anxiety, and behaviour change in relation to the swine flu outbreak: cross sectional telephone survey. BMJ..

[17] Bish A, Michie S (2010). Demographic and attitudinal determinants of protective behaviours during a pandemic: a review. Br J Health Psychol.

[18] Dryhurst S, Schneider C, Kerr J, Freeman A, Recchia G, van der Bles A, et al. Risk perceptions of COVID-19 around the world. J Risk Res. 2020:23(7-8):994-1006.

[19] Rogers RW (1975). A protection motivation theory of fear appeals and attitude change. J Psychol.

[20] Glanz K, Bishop D (2010). The role of behavioral science theory in development and implementation of public health interventions. Annu Rev Public Health.

[21] Rosenstock IM (1990). The health belief model: explaining health behavior through expectancies. Health behavior and health education. Theory, research, and practice.

[22] Ritvo P, Edwards SA, Glendon G, Mirea L, Knight JA, Andrulis IL (2012). Beliefs about optimal age and screening frequency predict breast screening adherence in a prospective study of female relatives from the Ontario site of the breast cancer family registry. BMC Public Health.

[23] Bashirian S, Barati M, Mohammadi Y, Moaddabshoar L, Dogonchi M (2019). An application of the protection motivation theory to predict breast self-examination behavior among female healthcare workers. Eur J Breast Health.

[24] Ogden J (2007). Health Psychology.

[25] Vandrevala T, Alidu L, Hendy J, Shafi S, Ala A. ‘It’s possibly made us feel a little more alienated’: how people from ethnic minority communities conceptualise COVID-19 and its influence on engagement with testing. J Health Serv Res Policy. 2022:13558196211054961. 10.1177/13558196211054961 PMC8948536 34978500

[26] Seedat F, Hargreaves S, Friedland J (2014). Engaging new migrants in infectious disease screening: a qualitative semi-structured interview study of UK migrant community health-care leads. PLoS One.

[27] Watson J, Whiting P, Brush JE (2020). Interpreting a covid-19 test result. BMJ..

[28] van der Pligt J (1996). Risk Perception and Self-Protective Behavior. Eur Psychol.

[29] Khademian F, Delavari S, Koohjani Z, Khademian Z. An investigation of depression, anxiety, and stress and its relating factors during COVID-19 pandemic in Iran. BMC Public Health. 2021;21(1):1-7. 10.1186/s12889-021-10329-3 PMC7856614 33535992

[30] Stainback K, Hearne B, Trieu M. COVID-19 and the 24/7 news cycle: does COVID-19 news exposure affect mental health? Socius: Sociological Research for a Dynamic World. 2020:6:1-15. 10.1177/2378023120969339.

[31] Scally G, Jacobson B, Abbasi K (2020). The UK’s public health response to covid-19. BMJ..

[32] Nguyen T, Wolff A (2021). Rapid diagnostics for SARS-CoV-2 virus: point-of-care testing and lessons learned during the pandemic. Bioanalysis..

