# Supplementary material for: Willingness of the UK public to volunteer for testing in relation to the COVID-19 pandemic
Source: BMC Public Health. 2022 Mar 22;22:565. doi: 10.1186/s12889-022-12848-z (PMC8938736; doi:10.1186/s12889-022-12848-z)
Supplement: Supplementary file 1 — Additional file 1. Questionnaire. Knowledge and Perceptions about Coronavirus items with scale points. Views on Testing for Coronavirus items with scale points. [file 12889_2022_12848_MOESM1_ESM.docx]

**Additional file 1 – Questionnaire**

**Attitudes on coronavirus testing during the pandemic**

Our team at Kingston University is undertaking a survey to find out about people’s perceptions and attitudes in relation to the testing for coronavirus during the pandemic.

This questionnaire is about the Coronavirus pandemic. For the purposes of this questionnaire certain definitions have been used to simplify information communication, in this study we will refer to the Coronavirus throughout, but we understand that COVID-19 is the disease and the disease-causing agent is the Coronavirus known as SARs-CoV2.

| **Questions** |  |  |  |  | |  |
| --- | --- | --- | --- | --- | --- | --- |
| Please create a random identifying word/number sequence.  Please take note of this word/number sequence because If you wish for your data to be removed after submitting your responses, we will require this from you. (Please try and ensure what you choose is something random including letters and numbers). | | | | | | |
| **Sex** | female | male | other | Rather not say | |  |
| **Age** | 18-24 | 25-30 | 31-40 | 41-50 | | 51-60 |
|  | 61-70 | 71+ |  |  | |  |
| **Highest level of educational qualification** | Post-graduate (eg PGCE, MSc PhD) | Graduate (eg BA/BSc Hons) | A-levels/BTEC level 3 | GCSE | | NVQ level 2 |
|  | none of the above | Other (please state) | | | | |
| **Ethnicity** | White  English, welsh, Scottish, northern Irish, British Irish, gypsy or traveler, any other Asian background | Black/African/Caribbean/black British,  African, Caribbean, any other black/African/Caribbean background | Asian or Asian British  Indian, Pakistani, Bangladeshi, Chinese, any other Asian background, black/ African/ Caribbean/black British | Mixed/ multiple ethnic group  White and black Caribbean, white and black African white and Asian, any other mixed background | | Other ethnic group  Arab  Any other ethnic group please state below |
| **Country of residence (Please state below)** |  | | | | | |
| **How long have you lived in your country of residence? (state number of years)** |  | | | | | |
| **Religion** | Christian | Muslim | Hindu | Sikh | | Buddhist |
|  | Judaism | No religion | | | Other (please state) | |
| **Occupation** | Unemployed | Employed | Furlough | student | | self-employed |
|  | Part time employed | retired |  |  | |  |
| Do you have any professional expertise in the following areas? | Health service | Social care | Scientist | Key worker | | None of the above |
| What do you consider your level of medical science knowledge to be? | Very advanced | advanced | average | poor | | Very poor |
| Do you consider yourself as having any form of disability (hidden or visible) or long-term illness? | YES | NO |  |  | |  |
| Have you been contacted directly by the government and considered to be in the “vulnerable” group. | YES | NO | Not Appropriate |  | |  |
| I have been tested for coronavirus | YES | NO |  |  | |  |
| If Yes, was the test result positive? | YES | NO |  |  | |  |
| I am certain I have had coronavirus but have not had a test | Strongly agree | agree | disagree | Strongly disagree | |  |
| Where have you picked up any information about coronavirus? (Tick all that apply) | News (newspaper/television/internet etc) | Social media (facebook/twitter etc) | General Practice/  Hospital | friends/  family | | other |
| Rank order the main ways in which coronavirus can be contracted or spread | Contract with Blood | Contact with vomit | Respiratory fluid droplets | Hand contact, including touching surfaces | | Contact with urine |
| What are the symptoms of coronavirus? (Tick all that are relevant) | new, Persistent Dry Cough | fever | loss of taste | headache | | sneezing |
|  | rash | loss of hearing | loss of smell |  | |  |

**Knowledge and perceptions about CORONAVIRUS**

| **Questions** | **Strongly**  **Disagree** | **Disagree** | **Agree** | **Strongly Agree** |
| --- | --- | --- | --- | --- |
| Coronavirus can affect all people |  |  |  |  |
| Coronavirus is more dangerous for people over 70 more than younger people |  |  |  |  |
| Coronavirus has greater effects on people with underlying health conditions |  |  |  |  |
| Coronavirus appears to effect ethnic minority communities more than others |  |  |  |  |
| Coronavirus effect men more than women |  |  |  |  |
| I think it is important to do what I can to protect myself from Coronavirus even if it effected my finances, business, work |  |  |  |  |
| I think it is important to do what I can to protect others from Coronavirus even if it effected my personal life. |  |  |  |  |
| It does not matter what I do, if I am going to get coronavirus it is meant to be |  |  |  |  |
| Coronavirus always makes you feel ill so if I had it I would know something is wrong |  |  |  |  |
| If I had Coronavirus I would worry people would think badly of me |  |  |  |  |
| Coronavirus can cause severe health problems for me |  |  |  |  |
| Coronavirus can cause severe health problems to others |  |  |  |  |
| Coronavirus poses a threat to my life |  |  |  |  |
| Coronavirus poses a threat to others |  |  |  |  |
| Coronavirus poses a threat to me |  |  |  |  |
| I won’t get coronavirus because I am fit and healthy |  |  |  |  |
| I won’t get coronavirus because I am young |  |  |  |  |
| I won’t get coronavirus because I am religious |  |  |  |  |
| I think we should prioritise returning to regular work/life patterns over extending the current “lockdown” to protect some lives |  |  |  |  |
| I closely follow news reports about the virus |  |  |  |  |
| I think that coronavirus is a very serious issue |  |  |  |  |
| I am worried that I will catch coronavirus |  |  |  |  |
| I am worried that family and friends with catch coronavirus |  |  |  |  |
| I am worried we won’t have enough food and water and other essential items during the outbreak |  |  |  |  |
| I am worried about missing work |  |  |  |  |
| I am worried about the amount of money we have coming in |  |  |  |  |
| I am worried about the long-term impacts this will have on my job prospects and the economy |  |  |  |  |
| I think coronavirus has had a positive impact on my life |  |  |  |  |
| I think coronavirus is likely to have a positive impact on society in the future |  |  |  |  |
| I am confident about my government’s response to the outbreak |  |  |  |  |

**Overall Views on Testing for Coronavirus**

| **Questions** | | **Strongly**  **Disagree** | **Disagree** | **Agree** | | **Strongly**  **Agree** |
| --- | --- | --- | --- | --- | --- | --- |
| Coronavirus testing is difficult because a new test needs to be developed |  | |  |  | |  |
| Coronavirus testing is important even if I feel well |  | |  |  | |  |
| Coronavirus testing is important if I display early signs of the illness |  | |  |  | |  |
| Coronavirus testing is important if I go into hospital with signs of the illness |  | |  |  | |  |
| Coronavirus testing is important if I live with vulnerable people |  | |  |  | |  |
| Coronavirus testing is important if I work with vulnerable people |  | |  |  | |  |
| Testing will tell me if I am immune |  | |  |  | |  |
| I think testing would protect me from getting coronavirus |  | |  |  | |  |
| I think testing would protect others from getting coronavirus |  | |  |  | |  |
| I think testing would protect healthcare workers from getting coronavirus |  | |  |  | |  |
| I think testing with allow me to know if I have previously had coronavirus |  | |  |  | |  |
| Testing as a way out of lockdown |  | |  |  | |  |
| I would consider getting myself tested for coronavirus |  | |  |  | |  |
| I would not consider being tested |  | |  |  | |  |
| I would pay to be tested |  | |  |  | |  |
| It is my doctor’s role to tell me whether I need testing |  | |  |  | |  |
| I trust my doctor to tell me if I need testing |  | |  |  | |  |
| I think I would know whether I need testing |  | |  |  | |  |
| I feel able to approach my doctor to ask for tests if I feel I need it |  | |  |  | |  |
| If I had coronavirus I would worry about being a burden to my family |  | |  |  | |  |
| If I was tested for coronavirus, I would be very worried about the result |  | |  |  | |  |
| If I was tested for coronavirus I would be relieved to know the results |  | |  |  | |  |
| If I was tested for coronavirus and did not have it, I would not need to take any further precautions |  | |  |  | |  |
| If I was tested for coronavirus and did not have it, I would need to be re-testing in the future |  | |  |  | |  |
| I don’t understand why testing (me/others) may be useful |  | |  |  | |  |
| Widespread testing across the country is now a waste of time and money |  | |  |  | |  |
| Widespread testing across the country is always a waste of time and money |  | |  |  | |  |
| It’s too late for widespread national testing to affect anything |  | |  |  | |  |
| I trust my government’s testing strategy | |  |  |  | |  |
| My government should have prioritized testing earlier in the outbreak |  | |  |  | |  |
| My government and its health advisors have clearly communicated the benefits of testing |  | |  |  | |  |
| My government and its health advisors have clearly communicated why widespread testing is difficult |  | |  |  | |  |
| I prefer testing in | community | | Hospital | | Home | |

**Coronavirus testing: PCR testing** (the current test available, which tests that the person actually has coronavirus):

| Questions | Strongly  Agree | Agree | Disagree | Strongly Disagree |
| --- | --- | --- | --- | --- |
| PCR testing is currently available in my country |  |  |  |  |
| PCR testing is used to test which people currently have coronavirus |  |  |  |  |
| A laboratory is needed to conduct this test |  |  |  |  |
| PCR testing is reliable for coronavirus |  |  |  |  |
| The results from PCR testing is available in a few hours |  |  |  |  |
| If a person has recovered from coronavirus, the PCR testing can tell if they have previously had the virus |  |  |  |  |
| PCR testing can be undertaken at home |  |  |  |  |
| PCR testing can be done only by specially trained scientists |  |  |  |  |
| PCR testing should only be provided for people who are showing signs of coronavirus infection |  |  |  |  |
| PCR testing should be available to everyone who thinks they have coronavirus, regardless of costs |  |  |  |  |
| Who should be prioritized for PCR testing (rank order)? | Frontline workers | key workers | vulnerable people | Family carers |
|  | General public |  |  |  |
|  | Social care | Patients in intensive care | people with suspected coronavirus | people living in care homes |
| The PCR test is not widely available because it is expensive to test everyone |  |  |  |  |
| The PCR test is not widely available because of the unavailable of key products required to make the test |  |  |  |  |
| Can you do the current PCR test on yourself? | Yes | NO |  |  |

**Coronavirus testing: Antibody testing (to look at whether person has had the coronavirus)**

| Questions | Strongly  Agree | Agree | Disagree | Strongly Disagree |
| --- | --- | --- | --- | --- |
| Antibody testing is currently available in my country |  |  |  |  |
| Antibody testing is used to test which people currently have coronavirus |  |  |  |  |
| A laboratory is needed to conduct this test |  |  |  |  |
| Antibody testing is reliable for coronavirus |  |  |  |  |
| The results from Antibody testing is available in a few hours |  |  |  |  |
| If a person has recovered from coronavirus, the Antibody testing can tell if they have previously had the virus |  |  |  |  |
| Antibody testing can be undertaken at home |  |  |  |  |
| Antibody testing can be done only by specially trained scientists |  |  |  |  |
| Antibody testing should only be provided for people who are showing signs of coronavirus infection |  |  |  |  |
| Antibody testing should be available to everyone who thinks they have coronavirus, regardless of costs |  |  |  |  |
| Who should be prioritized for Antibody testing (rank order)? | Frontline workers | key workers | vulnerable people | Family carers |
|  | General public |  |  |  |
|  | Social care | Patients in intensive care | people with suspected coronavirus | people living in care homes |
| The Antibody test is not widely available because it is expensive to test everyone |  |  |  |  |
| The Antibody test is not widely available because of the unavailable of key products required to make the test |  |  |  |  |
| Can you do the current Antibody test on yourself? | Yes | NO |  |  |

| What are most important ways in which the pandemic can be controlled? (Rank order) | Social distancing | Lockdown | Mass testing | Testing specific workers |
| --- | --- | --- | --- | --- |
|  | Testing patients | Testing vulnerable people | Vaccine development |  |

**THANK YOU FOR YOUR TIME IN COMPLETING THIS QUESTIONNAIRE**
