# Supplementary material for: Willingness of the UK public to volunteer for testing in relation to the COVID-19 pandemic
Source: BMC Public Health. 2022 Mar 22;22:565. doi: 10.1186/s12889-022-12848-z (PMC8938736; doi:10.1186/s12889-022-12848-z)
Supplement: Supplementary file 2 — Additional file 2. Factor Analysis. Table 3 and 4: Factors with loadings for Knowledge and perceptions about Coronavirus & Factors with loadings for Views on Testing for Coronavirus. [file 12889_2022_12848_MOESM2_ESM.docx]

**Factor analysis**

Factor analysis was carried out using SPSS v26 on the 29 items from the first part of the survey “Knowledge and Perceptions about Coronavirus” and then on the 32 items of the second part “Views on testing for coronavirus”, using a Varimax rotation method. Based upon eigen value analysis, 9 factors were retained for both scales. For the second (Testing) scale, any weakly-correlated items (<0.4) were removed from the relevant factor. Any items that loaded onto more than one factor were retained on the most highly correlated factor. Any factors with only one loaded item as well as items which did not load onto any factors were removed and kept as separate measurements. Analysis was then performed again with the final set of items. For the “Views on Testing for Coronavirus” part of the survey, this left 26 items separated across 8 factors which cumulatively explained 67.27% of variance, with eigen values 2.98, 2.94, 2.60, 2.53, 1.84, 1.67, 1.49, 1.45. For the first part of the survey (Knowledge and Perceptions about Coronavirus), the same process left a total of 26 items distributed across 8 factors, which cumulatively explained 62.60% of variance, with eigen values, 2.70, 2.56. 2.37, 1.96, 1.79, 1.73, 1.62, and 1.54. All factors with loadings are included in supplementary material. The item “I think Coronavirus is a very serious issue” was moved from factor 1 to factor 2, since it was a better logical fit to this factor and was weakly correlated with it (r=.404). In addition, factors 5 and 6 were combined due to the items being very similar. Reliability analysis was used to calculate internal consistency for these new combinations; factor 2 with the additional item from factor 1 demonstrated a relatively high reliability (α=.78), and the combination of factors 5 and 6 yielded acceptable reliability (α=.69). The factors derived from the “Views on Testing for Coronavirus” part of the survey were not altered.

**Table 3**

Factors with loadings for Knowledge and perceptions about Coronavirus

| **Item** | **1** | **2** | **3** | **4** | **5** | **6** | **7** | **8** |
| --- | --- | --- | --- | --- | --- | --- | --- | --- |
| I think it is important to do what I can to protect myself from Coronavirus even if it affected my finances, business, work | .723 |  |  |  |  |  |  |  |
| I think it is important to do what I can to protect others from Coronavirus even if it affected my personal life. | .678 |  |  |  |  |  |  |  |
| It does not matter what I do, if I am going to get coronavirus it is meant to be | -.547 |  |  |  |  |  |  |  |
| I think we should prioritize returning to regular work/life patterns overextending the current “lockdown” to protect some lives | -.626 |  |  |  |  |  |  |  |
| I think that coronavirus is a very serious issue | .513 |  |  |  |  |  |  |  |
| Coronavirus can cause severe health problems for me |  | .818 |  |  |  |  |  |  |
| Coronavirus can cause severe health problems for others |  | .644 |  |  |  |  |  |  |
| Coronavirus poses a threat to others |  | .643 |  |  |  |  |  |  |
| Coronavirus poses a threat to me |  | .764 |  |  |  |  |  |  |
| I won’t get coronavirus because I am fit and healthy |  |  | .822 |  |  |  |  |  |
| I won’t get coronavirus because I am young |  |  | .842 |  |  |  |  |  |
| I won’t get coronavirus because I am religious |  |  | .665 |  |  |  |  |  |
| I am worried about missing work |  |  |  | .692 |  |  |  |  |
| I am worried about the amount of money we have coming in |  |  |  | .814 |  |  |  |  |
| I am worried about the long-term impacts this will have on my job prospects and the economy |  |  |  | .779 |  |  |  |  |
| Coronavirus appears to effect ethnic minority communities more than others |  |  |  |  | .853 |  |  |  |
| Coronavirus effects men more than women |  |  |  |  | .837 |  |  |  |
| Coronavirus can affect all people |  |  |  |  |  | .494 |  |  |
| Coronavirus is more dangerous for people over 70 more than younger people |  |  |  |  |  | .817 |  |  |
| Coronavirus has greater effects on people with underlying health conditions |  |  |  |  |  | .791 |  |  |
| I think coronavirus has had a positive impact on my life |  |  |  |  |  |  | .861 |  |
| I think coronavirus is likely to have a positive impact on society in the future |  |  |  |  |  |  | .877 |  |
| If I had Coronavirus, I would worry people would think badly of me |  |  |  |  |  |  |  | .421 |
| I am worried that I will catch coronavirus |  |  |  |  |  |  |  | .685 |
| I am worried that family and friends with catch coronavirus |  |  |  |  |  |  |  | .514 |
| I am worried we won’t have enough food and water and other essential items during the outbreak |  |  |  |  |  |  |  | .604 |

**Table 4**

Factors with loadings for Views on Testing for Coronavirus

| **Item** | **1** | **2** | **3** | **4** | **5** | **6** | **7** | **8** |
| --- | --- | --- | --- | --- | --- | --- | --- | --- |
| I don’t understand why testing (me and others) for coronavirus may be useful | .698 |  |  |  |  |  |  |  |
| Widespread coronavirus testing across the country is now a waste of time and money | .844 |  |  |  |  |  |  |  |
| Widespread coronavirus testing across the country is always a waste of time and money | .823 |  |  |  |  |  |  |  |
| It’s too late for widespread national coronavirus testing to affect anything | .787 |  |  |  |  |  |  |  |
| Coronavirus testing is important even if I feel well |  | .443 |  |  |  |  |  |  |
| Coronavirus testing is important if I display early signs of the illness |  | .615 |  |  |  |  |  |  |
| Coronavirus testing is important if I go into hospital with signs of the illness |  | .757 |  |  |  |  |  |  |
| Coronavirus testing is important if I live with vulnerable people |  | .854 |  |  |  |  |  |  |
| Coronavirus testing is important if I work with vulnerable people |  | .833 |  |  |  |  |  |  |
| I think widespread testing would protect me from getting Coronavirus |  |  | .861 |  |  |  |  |  |
| I think widespread testing would protect others from getting coronavirus |  |  | .858 |  |  |  |  |  |
| I think testing would protect healthcare workers from getting coronavirus |  |  | .803 |  |  |  |  |  |
| Testing is a way out of lockdown |  |  | .453 |  |  |  |  |  |
| I trust my governments coronavirus testing strategy |  |  |  | .812 |  |  |  |  |
| My government should have prioritized testing for coronavirus earlier in the outbreak |  |  |  | -.590 |  |  |  |  |
| My government and its health advisors have clearly communicated the benefits of testing for coronavirus |  |  |  | .849 |  |  |  |  |
| My government and its health advisors have clearly communicated why widespread testing for coronavirus is difficult |  |  |  | .807 |  |  |  |  |
| I would consider getting myself testing for coronavirus |  |  |  |  | .796 |  |  |  |
| I would not consider being tested for Coronavirus |  |  |  |  | -.792 |  |  |  |
| It is my doctor’s role to tell me whether I need testing for Coronavirus |  |  |  |  |  | .738 |  |  |
| I trust my doctor to tell me if I need testing for coronavirus |  |  |  |  |  | .831 |  |  |
| I feel able to approach my doctor to ask for tests for coronavirus, if I feel I need it |  |  |  |  |  | .597 |  |  |
| Testing will tell me if I am immune |  |  |  |  |  |  | .802 |  |
| I think testing will allow me to know if I have previously had coronavirus |  |  |  |  |  |  | .793 |  |
| If I had coronavirus, I would worry about being a burden to my family |  |  |  |  |  |  |  | .800 |
| If I was tested for coronavirus, I would be very worried about the result |  |  |  |  |  |  |  | .797 |
