# Supplementary material for: Willingness of the UK public to volunteer for testing in relation to the COVID-19 pandemic
Source: BMC Public Health. 2022 Mar 22;22:565. doi: 10.1186/s12889-022-12848-z (PMC8938736; doi:10.1186/s12889-022-12848-z)
Supplement: Supplementary file 3 — Additional file 3. Table 2. Means, standard deviations, Cronbach’s alpha scores and list of items, for each subscale. [file 12889_2022_12848_MOESM3_ESM.docx]

**Table 2**

Means, standard deviations, Cronbach’s alpha scores and list of items, for each subscale.

| Subscale | Mean (SD) | Cronbach’s Alpha reliability score (α) | Items |
| --- | --- | --- | --- |
| Part 1: Knowledge and perceptions about Coronavirus | | | |
| 1. Confidence that taking action is effective  Number of items 4 | 13.74 (1.92) | .625 | I think it is important to do what I can to protect myself from Coronavirus even if it affected my finances, business, work  I think it is important to do what I can to protect others from Coronavirus even if it affected my personal life.  It does not matter what I do, if I am going to get coronavirus it is meant to be *  I think we should prioritize returning to regular work/life patterns over extending the current “lockdown” to protect some lives * |
| 2. Perceived Severity and Threat  Number of items 5 | 17.51  (2.18) | .793 | I think that coronavirus is a very serious issue  Coronavirus can cause severe health problems for me  Coronavirus can cause severe health problems for others  Coronavirus poses a threat to others  Coronavirus poses a threat to me |
| 3. Personal Susceptibility  Number of items 3 | 3.83  (1.16) | .776 | I won’t get coronavirus because I am fit and healthy  I won’t get coronavirus because I am young  I won’t get coronavirus because I am religious |
| 4.Worry about economic implications  Number of items 3 | 7.15  (2.09) | .696 | I am worried about missing work  I am worried about the amount of money we have coming in  I am worried about the long-term impacts this will have on my job prospects and the economy |
| 5.Impact of coronavirus on specific demographic groups  Number of items 5 | 16.88  (2.27) | .686 | Coronavirus appears to effect ethnic minority communities more than others  Coronavirus effects men more than women  Coronavirus can effect all people  Coronavirus is more dangerous for people over 70 more than younger people  Coronavirus has greater effects on people with underlying health conditions |
| 6.Positive Impacts on self and society  Number of items 2 | 4.35  (1.36) | .717 | I think coronavirus has had a positive impact on my life  I think coronavirus is likely to have a positive impact on society in the future |
| 7. Worry about the Health and Social Impacts on Self and Family  Number of items 4 | 9.61  (1.82) | .452 | If I had Coronavirus, I would worry people would think badly of me  I am worried that I will catch coronavirus  I am worried that family and friends with catch coronavirus  I am worried we won’t have enough food and water and other essential items during the outbreak |
| Part 2: Views on Testing for Coronavirus | | | |
| 1.Negative views about widespread testing  Number of items 4 | 6.03  (2.15) | .847 | I don’t understand why testing (me and others) for coronavirus may be useful  Widespread coronavirus testing across the country is now a waste of time and money  Widespread coronavirus testing across the country is always a waste of time and money  It’s too late for widespread national coronavirus testing to affect anything |
| 2. Importance of Testing “by need”  Number of items 5 | 17.70  (2.14) | .814 | Coronavirus testing is important even if I feel well  Coronavirus testing is important if I display early signs of the illness  Coronavirus testing is important if I go into hospital with signs of the illness  Coronavirus testing is important if I live with vulnerable people  Coronavirus testing is important if I work with vulnerable people |
| 3.Testing considered as an effective protective measure  Number of items 4 | 10.87  (2.58) | .804 | I think widespread testing would protect me from getting Coronavirus  I think widespread testing would protect others from getting coronavirus  I think testing would protect healthcare workers from getting coronavirus  Testing is a way out of lockdown |
| 4.Trust in Government approach to testing  Number of items 4 | 8.55  (2.48) | .795 | I trust my governments coronavirus testing strategy  My government should have prioritized testing for coronavirus earlier in the outbreak *  My government and its health advisors have clearly communicated the benefits of testing for coronavirus  My government and its health advisors have clearly communicated why widespread testing for coronavirus is difficult |
| 5.Willingness to be tested  Number of items 2 | 6.60  (1.26) | .814 | I would consider getting myself testing for coronavirus  I would not consider being tested for Coronavirus * |
| 6.Trust in doctor’s advice about testing  Number of items 3 | 7.51  (1.61) | .576 | It is my doctor’s role to tell me whether I need testing for Coronavirus  I trust my doctor to tell me if I need testing for coronavirus  I feel able to approach my doctor to ask for tests for coronavirus, if I feel I need it |
| 7.Beliefs that Testing provides immunity  Number of items 2 | 4.52  (1.36) | .554 | Testing will tell me if I am immune  I think testing will allow me to know if I have previously had coronavirus |
| 8.Worries about testing outcome  Number of items 2 | 5.15  (1.43) | .556 | If I had coronavirus, I would worry about being a burden to my family  If I was tested for coronavirus, I would be very worried about the result |

*****items reverse coded
